# Prevalence and Antimicrobial Susceptibility Pattern of Methicillin-resistant *Staphylococcus Aureus* [MRSA] Isolates at a Tertiary Care Hospital in Mangalore, South India

**DOI:** 10.4103/0974-2727.72155

**Published:** 2010

**Authors:** Vidya Pai, Venkatakrishna I Rao, Sunil P Rao

**Affiliations:** Department of Microbiology, Yenepoya Medical College, Nithyananda Nagar, Mangalore – 575 018, India

**Keywords:** Antibiotic, MRSA, prevalence

## Abstract

**Background/Aim:**

Methicillin-resistant *Staphylococcus aureus* (MRSA) is an important cause of nosocomial infections worldwide. The aim of this study was to determine the prevalence of MRSA and their antimicrobial susceptibility pattern in our hospital located in Mangalore, India.

**Materials and Methods:**

The bacterial isolates from various clinical specimens of patients admitted in our hospital were cultured as per standard protocol and all isolates of *Staphylococcus aureus* obtained were included in the study. The isolates were identified by standard methods like catalase test, slide and tube coagulase tests, and growth on Mannitol salt agar (HiMedia Lab, Mumbai). The antimicrobial susceptibility testing was performed by Kirby–Bauer disc diffusion method. The D-test for inducible clindamycin resistance was performed. The isolates were tested for methicillin resistance by using oxacillin disc by disc diffusion method and confirmed by agar screen test (oxacillin 6 μgm/ml). The results were interpreted according to CLSI criteria.

**Results:**

During a period of one year, a total of 237 isolates of *S. aureus* were studied and 69 (29.1%) were found to be methicillin-resistant. MRSA isolates showed greater resistance to multiple drugs than methicillin sensitive Staphylococcus aureus MSSA isolates. Inducible clindamycin resistance was 18.8% in MRSA as against 3.5% in MSSA. About 40–50% of MRSA were resistant to erythromycin, gentamicin, and chloramphenicol, while less than 30% were resistant to ciprofloxacin and amikacin. However, all strains were sensitive to vancomycin.

**Conclusion:**

The regular surveillance of hospital-acquired infections of MRSA may be helpful in formulating and monitoring the antibiotic policy. This may also help in preserving antibiotics like vancomycin, only for life-threatening staphylococcal diseases.

## INTRODUCTION

After the emergence of MRSA as a nosocomial pathogen in the early 1960s, there have been an increasing number of outbreaks of MRSA infections in hospitals reported from many countries. Life-threatening sepsis, endocarditis, and osteomyelitis caused by MRSA have also been reported.[1] Since resistance to multiple antibiotics among MRSA isolates is very common, there is a possibility of extensive outbreaks, which may be difficult to control. MRSA is now one of the commonest nosocomial pathogens, and asymptomatically colonized healthcare workers are the major sources of MRSA in the hospital environment. Early detection of MRSA and formulation of effective antibiotic policy in tertiary care hospitals is of paramount importance from the epidemiological point. The present study has been carried out in our medical college hospital with an aim to know the prevalence and antibiotic sensitivity pattern of *Staphylococcus aureus* isolates, in order to utilize the information obtained and formulate antibiotic policy and appropriate control measures.

## MATERIALS AND METHODS

This study was carried out in our teaching hospital at Mangalore, India from June 2007 to June 2008. A total of 237 isolates of *S. aureus* were included in the study. These strains were obtained from various clinical samples like pus, sputum, urine, blood, and body fluids from the inpatients of our hospital. The specimens were cultured on blood agar and MacConkey agar plates and incubated aerobically at 37°C for 48 hours. The isolates were identified using standard tests like catalase, slide and tube coagulase, and growth on Mannitol salt agar.[2] Antibiotic sensitivity testing was performed by Kirby–Bauer disc diffusion method for the following antibiotics: amikacin (30 μgm), ciprofloxacin (5 μgm), chloramphenicol (30 μgm), clindamycin (2 μgm), gentamicin (10 μgm), erythromycin (15 μmg), netilmicin (30 μgm), penicillin (10 units), rifampicin (5 μgm), and vancomycin (30 μmg). The erythromycin (15 μgm) disc was placed at a distance of 15 mm (edge-to-edge) from clindamycin (2 μgm) disc on a Mueller–Hinton agar plate previously inoculated with 0.5 McFarland bacterial suspension. Following overnight incubation at 37°C, flattening of zone (D-shaped) around clindamycin in the area between the two discs, indicated inducible clindamycin resistance.

Test for methicillin resistance was performed by Kirby–Bauer disc diffusion method using oxacillin (1 μgm) disc on Mueller–Hinton agar (HiMedia Labs, Mumbai) with 24 hours incubation at 35°C. Results were interpreted according to the criteria of CLSI.[3] Methicillin resistance was confirmed by agar screen test using Mueller–Hinton agar plate supplemented with 4% NaCl and oxacillin (6 μgm/ml). *Staphylococcus aureus* NCTC 6571 was used as a control methicillin-sensitive strain and *S. aureus* NCTC 12493 as a control methicillin-resistant strain.

## RESULTS

**Figure 1:**
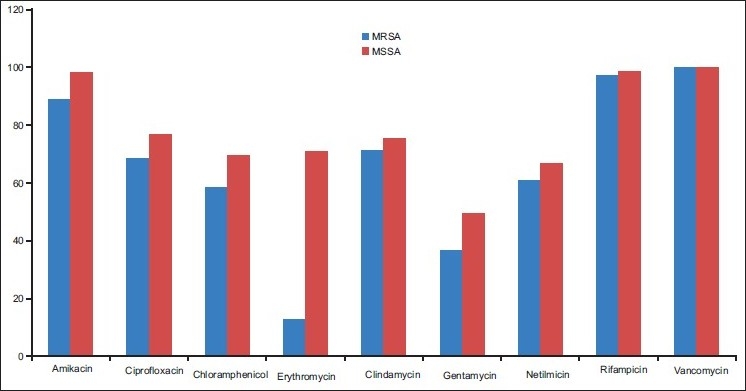
Frequency in percentage of sensitive stains of MRSA and MSSA

A total of 237 isolates of *S. aureus* were obtained from different clinical samples from inpatients of the hospital. Pus and wound swabs accounted for the majority of isolates that is 181 (76.3%), followed by urine, respiratory specimen, and blood and body fluids. Methicillin resistance was documented in 69 (29.1%) of 237 isolates. The distribution of 69 MRSA isolates in relation to various specimens is provided in Table 1. The sensitivity data of MRSA and MSSA is shown in Figure 1. All strains of MSSA were found to be resistant to penicillin. A total of 109 (45.9%) isolates were resistant to erythromycin (60 MRSA and 49 MSSA isolates). Among erythromycin resistant Strains of MRSA, 40 were sensitive to clindamycin, 13 (18.8%) showed positive D-test (inducible clindamycin resistance), and seven were resistant to both erythromycin and clindamycin. Out of 168 isolates of MSSA, 49 were resistant to erythromycin. Inducible clindamycin resistance was shown by only 6 (3.5%) isolates of MSSA.

**Table 1:** Frequency of *S. aureus* and MRSA in specimens

Clinical specimen	*S. aureus* (*n* = 237)	MRSA (*n* = 69)	Percentage
Pus and wound swab	181	49	27.07
Blood	09	02	22.22
Respiratory sample	17	05	29.40
Urine	28	12	42.80
Body fluids	02	01	50.00

## DISCUSSION

The prevalence rate of MRSA was found to be 29.1% in our study, which is in accordance with investigators from India (32.8%)[4] and Nepal (26.14%).[5] On the contrary, some studies have reported alarmingly high incidence of MRSA infection. The epidemiology of MRSA over different parts of India is not uniform. Reports from a Delhi hospital showed a prevalence rate of 51.6% in 2001, whereas it was reported as 38.44% in the same hospital in 2008.[6] Another study from north India has reported an incidence comparable to our study.[7]

MRSA are often multidrug-resistant. Currently, the majority of *S. aureus* strains in communities are beta-lactamase producers, hence resistant to penicillin and ampicillin. A study from Maharashtra has reported that more than 90% isolates from South Maharashtra have been found resistant to penicillin, ampicillin, erythromycin, gentamicin, and tobramycin, whereas only 39.1% were resistant to methicillin.[8] Our study also showed a high degree of resistance to erythromycin, gentamicin, and chloramphenicol. Hence, the choice is obviously oxacillin. However, fearing MRSA, clinicians may exploit vancomycin, especially when a sensitivity study is not performed. The present study reports that antibiotics other than vancomycin, for instance, clindamycin, amikacin, ciprofloxacin, and netilmicin can be promising if a susceptibility testing is done, reserving vancomycin for life-threatening infections. Similar findings have been reported from other studies as well.[8,9]

Multidrug resistance among MRSA strains was higher than those that were sensitive to methicillin. Ciprofloxacin was proposed to be an alternate therapy for MRSA infection.[10] Although rapidly developing resistance to ciprofloxacin has been reported,[11] the antibiotic was found working on *S. aureus* (68.2%) in our hospital. This is perhaps due to the differential clonal expansion and drug pressure in the community.

It was also observed that percentage of inducible clindamycin resistance was higher amongst MRSA (18.8%) as compared to MSSA (3.5%). This was in concordance with a few of the studies reported earlier – inducible resistance of 24.4% in MRSA and 14.8% in MSSA;[12] 30% in MRSA and 10% in MSSA.[13] Another study from India showed very high frequency of inducible resistance (63%) in erythromycin-resistant; clindamycin-sensitive isolates being 74% in MRSA and 45% in MSSA.[14] In the light of the restricted range of antibiotics available for the treatment of methicillin-resistant staphylococcal infections and the known limitations of vancomycin, clindamycin should be considered for the management of serious soft-tissue infections with methicillin-resistant staphylococci that are sensitive to clindamycin.

The higher price of vancomycin, its unavailability in many parts of the country, and also the possibility of emergence of resistance to the drug should at least make the clinicians look into the alternatives. Therefore, regular surveillance of hospital-associated infections including antimicrobial susceptibility pattern of MRSA and formulation of a definite antibiotic policy may be helpful in reducing the burden of MRSA infections in the hospital.

## References

[1] Cox RA, Conquest C, Mallaghan C, Marples RR (1995). A major outbreak of methicillin resistant *Staphylococcus aureus* caused by a new phage type (EMRSA-16). J Hosp Infect.

[2] Collee JG, Miles RS, Watt B, Mackie, McCartney (1996). Test for the identification of bacteria. Practical Medical Microbiology.

[3] (2007). Performance Standards for Antimicrobial susceptibility testing. Seventeenth informational supplement. Clinical Laboratory Standards Institute.

[4] Mehta AP, Rodrigue C, Sheth K, Jani S, Hakimiyan A, Fazalbhoy (1998). Control of methicillin resistant *Staphylococcus aureus* in a tertiary care center. A five year studyJ Med Microbiol.

[5] Kumari N, Mohapatra TM, Singh YI (2008). Prevalence of methicillin resistant Staphylococcus aureus [MRSA] in a tertiary care hospital in Eastern Nepal. JNMA J Nepal Med Assoc.

[6] Tiwari HK, Sapkota D, Sen MR (2008). High prevalence of multidrug-resistant MRSA in a tertiary care hospital of northern India. Infection and Drug Resistance.

[7] Mohanty S, Kapil A, Dhawan B (2004). Bacteriological and antimicrobial susceptibility profile of soft tissue infections from Northern India. Indian J Med Microbiol.

[8] Kandle SK, Ghatole MP, Takpere AY, Hittinhalli VB, Yemul VL (2003). Bacteriophage typing and antibiotic sensitivity pattern of *Staphylococcus aureus* from clinical specimen in and around Solapur (South Maharashtra). J Commun Dis.

[9] Agnihotri N, Kaistha N, Gupta V (2004). Antimicrobial susceptibility of isolates form neonatal septicemia. Jpn J Infect Dis.

[10] Sharon MS, Robert HK, Flor TT (1989). Ciprofloxacin in therapy for *Methicillin Resistant Staphylococcus aureus* infections or colonizations. Antimicrob Agents Chemother.

[11] Blumberg HM, Rimland D, Carroll DJ, Terry P, Wachsmuth IK (1991). Rapid development of Ciprofloxacin resistance in *methicillin sensitive and methicillin resistant S.aureus*. J Infect Dis.

[12] Yilmaz G, Aydin K, Iskender S, Caylan R, Koksal I (2007). Detection and prevalence of inducible clindamycin resistance in staphylococci. J Med Microbiol.

[13] Gadepalli R, Dhawan B, Mohanty S, Kapil A, Das BK, Chaudhry R (2006). Inducible clindamycin resistance in clinical isolates of *Staphylococcus aureus*. Indian J Med Res.

[14] Ajantha GS, Kulkarni RD, Shetty J, Shubhada C, Jain P (2008). Phenotypic detection of inducible clindamycin resistance amongst *Staphylococcus aureus* isolates by using lower limit of recommended inter-disk distance. Indian J Pathol Microbiol.

